# Kappa Free Light Chains and IgG Combined in a Novel Algorithm for the Detection of Multiple Sclerosis

**DOI:** 10.3390/brainsci10060324

**Published:** 2020-05-27

**Authors:** Monika Gudowska-Sawczuk, Joanna Tarasiuk, Alina Kułakowska, Jan Kochanowicz, Barbara Mroczko

**Affiliations:** 1 Department of Biochemical Diagnostics, Medical University of Bialystok, Waszyngtona 15A St., 15-269 Bialystok, Poland; mroczko@umb.edu.pl; 2 Department of Neurology, Medical University of Bialystok, M. Skłodowskiej—Curie 24A St., 15-276 Bialystok, Poland; amirtarasiuk@wp.pl (J.T.); alakul@umb.edu.pl (A.K.); kochanowicz@vp.pl (J.K.); 3 Department of Neurodegeneration Diagnostics, Medical University of Bialystok, Waszyngtona 15A St., 15-269 Bialystok, Poland

**Keywords:** multiple sclerosis, diagnostic markers, immunoglobulins, kappa, free light chains

## Abstract

Background: It is well known that the cerebrospinal fluid (CSF) concentrations of free light chains (FLC) and immunoglobulin G (IgG) are elevated in multiple sclerosis patients (MS). Therefore, in this study we aimed to develop a model based on the concentrations of free light chains and IgG to predict multiple sclerosis. We tried to evaluate the diagnostic usefulness of the novel κIgG index and λIgG index, here presented for the first time, and compare them with the κFLC index and the λFLC index in multiple sclerosis patients. Methods: CSF and serum samples were obtained from 76 subjects who underwent lumbar puncture for diagnostic purposes and, as a result, were divided into two groups: patients with multiple sclerosis (*n* = 34) and patients with other neurological disorders (control group; *n* = 42). The samples were analyzed using turbidimetry and isoelectric focusing. The κIgG index, λIgG index, κFLC index, and λFLC index were calculated using specific formulas. Results: The concentrations of CSF κFLC, CSF λFLC, and serum κFLC and the values of κFLC index, λFLC index, and κIgG index were significantly higher in patients with multiple sclerosis compared to controls. CSF κFLC concentration and the values of κFLC index, λFLC index, and κIgG index differed in patients depending on their pattern type of oligoclonal bands. κFLC concentration was significantly higher in patients with pattern type 2 and type 3 in comparison to those with pattern type 1 and type 4. The κFLC index, λFLC index, and κIgG index were significantly higher in patients with pattern type 2 in comparison to those with pattern type 4. The κFLC index and κIgG index were significantly higher in patients with pattern type 2 in comparison to those with pattern type 1, and in patients with pattern type 3 compared to those with pattern type 4. The κIgG index was markedly elevated in patients with pattern type 3 compared to those with pattern type 1. In the total study group, κFLC, λFLC, κFLC index, λFLC index, κIgG index, and λIgG index correlated with each other. The κIgG index showed the highest diagnostic power (area under the curve, AUC) in the detection of multiple sclerosis. The κFLC index and κIgG index showed the highest diagnostic sensitivity, and the κIgG index presented the highest ability to exclude multiple sclerosis. Conclusion: This study provides novel information about the diagnostic significance of four markers combined in the κIgG index. More investigations in larger study groups are needed to confirm that the κIgG index can reflect the intrathecal synthesis of immunoglobulins and may improve the diagnosis of multiple sclerosis.

## 1. Introduction

Multiple sclerosis (MS) is a common neuroinflammatory and neurodegenerative disorder of the central nervous system (CNS) [1]. The etiology of multiple sclerosis is still unknown. However, the major pathology is mediated by an auto-reactive immune process of multifocal myelin destruction throughout the CNS. Prompt and accurate diagnosis is particularly important for the clinical management of patients, since disease-modifying therapies are the most effective at the early stage of the disease [2,3]. A perfect biomarker should allow the early diagnosis of a disease, aid in determining the prognosis of a disease, and be rapid and easily testable. Currently, there is no specific test for the diagnosis of multiple sclerosis. According to the 2017 revisions of the McDonald diagnostic criteria for MS, the diagnosis of this disease is based on clinical symptoms, imaging by MRI technology, and laboratory testing including cerebrospinal fluid (CSF) examination [4].

The main feature of multiple sclerosis consists of abnormalities of the cellular and humoral immune system. Combined actions of B cells and T cells play a role in the full development of demyelination and in the secretion of immunoglobulins. Therefore, in more than 90% of patients, an elevated level of immunoglobulins synthesized in the intrathecal space can be observed, and IgG oligoclonal bands (OCBs) are detected in the CSF. However, there is a proportion of subjects, i.e., patients presenting with their first episode of multiple sclerosis, whose results of oligoclonal bands are negative. On the other hand, increased intrathecal immunoglobulin synthesis may occur also in other inflammatory CNS disorders, and therefore, this test is not specific for MS [5,6,7,8].

It is well known that human immunoglobulins are composed of two heavy and two light chains. There are two types of light chains, kappa (κ) and lambda (λ), that are produced by B lymphocytes during the synthesis of immunoglobulins. Physiologically, an excess of light chains is normally produced. These light chains that are not combined with heavy chains are called free light chains (FLC). It has been proven that B cell abnormalities are associated with disorders leading to an abnormal concentration of free light chains [9,10]. Therefore, in this study we aimed to develop a model based on free light chains and other available laboratory data to predict multiple sclerosis. We tried to evaluate the diagnostics usefulness of the novel κIgG index and λIgG index and compare them to the already known κFLC index and λFLC index used for the assessment of patients with MS. 

## 2. Material and Methods

### 2.1. Subjects

**Figure 1:**
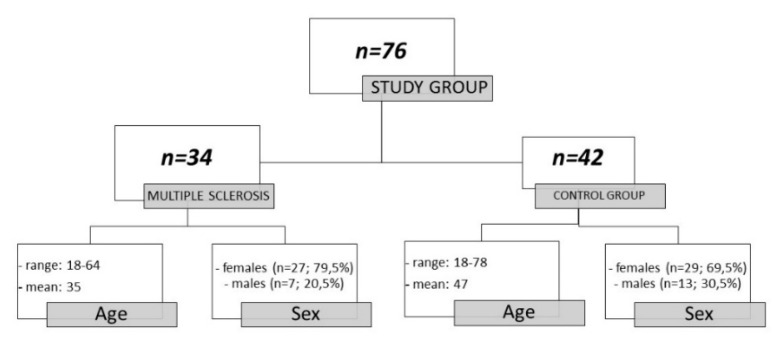
Characteristics of the study group.

This study was approved by the Bioethical Committee of the Medical University of Bialystok. Informed consent was obtained from all individuals included in the study. The patients were admitted to the Department of Neurology at the Medical University of Bialystok and underwent lumbar puncture for diagnostic purposes. Paired CSF and serum samples from the patients were collected between 2018 and 2020. The tested group consisted of 76 patients with neurological disorders who were divided into 2 subgroups: relapsing–remitting MS patients (*n* = 34) and a control group (*n* = 42) (Figure 1). All MS patients included in the study were in the process of receiving an MS diagnosis. They had a history of one clinical attack, and there was no evidence of dissemination in time according to magnetic resonance imaging (MRI). Finally, after CSF analysis which revealed OCBs presence, they were diagnosed with relapsing–remitting multiple sclerosis according to MacDonald criteria 2017 [4]. The degree of neurological impairment in patients diagnosed with multiple sclerosis from whom CSF was obtained was evaluated using the expanded disability status scale [11]. All evaluations rated between 1 and 2 points, indicating an early stage of the disease. All MS patients were not treated with any disease-modifying drugs or glucocorticosteroids at the time of lumbar puncture. The control group (29 females and 13 males; age range: 18–78 years) included patients eventually diagnosed with multifocal vascular lesions of the CNS (*n* = 18), discopathy (*n* = 6), idiopathic cephalgia (*n* = 9), dementia (*n* = 3), idiopathic (Bell’s) facial nerve palsy (*n* = 3), epilepsy (*n* = 1), herpetic encephalitis (*n* = 1), hydrocephalus (*n* = 1). Out of 34 patients with multiple sclerosis, 31 had OCBs in the CSF but not in serum (pattern type 2), and 3 had OCBs in CSF and serum, with additional OCBs in the CSF (pattern type 3). Out of 42 patients in the control group, 21 had no bands in CSF and serum (pattern type 1), 4 had pattern type 3, 16 had identical OCBs in CSF and serum (pattern type 4), and 1 had monoclonal bands in CSF and serum (pattern type 5). 

### 2.2. Sample Collection

CSF specimens were collected from each patient by lumbar puncture. The samples were collected into polypropylene tubes, centrifuged, aliquoted, and frozen at −80 °C until assayed. Venous blood samples were collected and centrifuged to separate the serum. The serum samples were aliquoted and frozen at −80 °C until assayed.

IgG oligoclonal bands determination in human CSF and serum was performed at the time of diagnosis using isoelectric focusing on agarose gel. Each patient’s serum and CSF samples were analyzed in parallel, in order to compare the IgG distribution. According to the manufacturer’s instructions, the assay includes two steps. Firstly, we performed isoelectrofocusing on agarose gel to fractionate the proteins in the CSF and serum med. Secondly, we carried out immunofixation with peroxidase-labelled anti-IgG antiserum to detect IgG oligoclonal bands and demonstrate the distribution of IgG in both fluids (Hydragel 3 CSF Isofocusing; Hydrasys; Sebia). The concentrations of κFLC, λFLC, albumin, IgG, IgM, and IgA in CSF and serum were measured according to the turbidimetric method (Optilite; The Binding Site). The κIgG index, λIgG index, κFLC index, and λFLC index were calculated according to the following formulas: $\frac{\mathrm{CSF}\;\kappa \mathrm{FLC}\;\left(\frac{\mathrm{mg}}{L}\right)/\mathrm{serum}\;\kappa \mathrm{FLC}\left(\frac{\mathrm{mg}}{L}\right)}{\mathrm{CSF}\;\mathrm{IgG}\;\left(\frac{\mathrm{mg}}{L}\right)/\mathrm{serum}\;\mathrm{IgG}\left(\frac{g}{L}\right)}\times 100$, $\frac{\mathrm{CSF}\;\lambda \mathrm{FLC}\;\left(\frac{\mathrm{mg}}{L}\right)/\mathrm{serum}\;\lambda \mathrm{FLC}\left(\frac{\mathrm{mg}}{L}\right)}{\mathrm{CSF}\;\mathrm{IgG}\;\left(\frac{\mathrm{mg}}{L}\right)/\mathrm{serum}\;\mathrm{IgG}\left(\frac{g}{L}\right)}\times 100$, $\frac{\mathrm{CSF}\;\kappa \mathrm{FLC}\left(\frac{\mathrm{mg}}{L}\right)/\mathrm{serum}\;\kappa \mathrm{FLC}\left(\frac{\mathrm{mg}}{L}\right)}{\mathrm{CSF}\;\mathrm{albumin}\left(\frac{\mathrm{mg}}{L}\right)/\mathrm{serum}\;\mathrm{albumin}\left(\frac{\mathrm{mg}}{L}\right)}$ and $\frac{\mathrm{CSF}\;\lambda \mathrm{FLC}\left(\frac{\mathrm{mg}}{L}\right)/\mathrm{serum}\;\lambda \mathrm{FLC}\left(\frac{\mathrm{mg}}{L}\right)}{\mathrm{CSF}\;\mathrm{albumin}\left(\frac{\mathrm{mg}}{L}\right)/\mathrm{serum}\;\mathrm{albumin}\left(\frac{\mathrm{mg}}{L}\right)}$, respectively. In cases of FLCs concentrations below the lower limit of detection, we used the corresponding detection limit (CSF κFLC, 0.30 mg/L, CSF λFLC, 0.65 mg/L). Intrathecal synthesis was also evaluated using albumin, IgG, IgA, and IgM quotients (Q_Alb_, Q_IgG_, Q_IgA_, Q_IgM_, respectively). 

### 2.3. Statistical Analysis

Data were stored and analyzed in Statistica 13.3. Differences between the multiple sclerosis and the control group were evaluated by Mann–Whitney U test. To test the hypothesis about the differences between subgroups, ANOVA rank Kruskal–Wallis test was performed. The post-hoc test was applied to determine which groups were different. We considered *p*-values < 0.05 as statistically significant. The diagnostic performance of each test was calculated as sensitivity, specificity, positive predictive value (PPV), negative predictive value (NPV), and accuracy (ACC). We used the area under the receiver operating characteristic (AUC ROC) curve to determine the optimal cut-off value and to calculate the diagnostic performance of the tests.

## 3. Results

The results of routine laboratory tests for patients with MS and the control group are presented in Table 1. Statistically significant differences between MS and controls in the Mann–Whitney U test were observed for the concentration of serum albumin and serum and CSF IgM and the values of Q_IgM_ and Q_IgG_ (*p* = 0.010; *p* = 0.047; *p* = 0.003; *p* = 0.002; *p* = 0.002, respectively).

### 3.1. CSF and Serum Concentrations of κFLC and λFLC

We determined the concentrations of κFLC and λFLC in the CSF and serum. κFLC and λFLC concentrations in the CSF and serum κFLC concentration were markedly elevated in MS patients (3.050 mg/L, 2.050 mg/L, 13.480 mg/L, respectively) compared to controls (0.310 mg/L, *p* < 0.001; 0.720 mg/L, *p* = 0.017; 16.265 mg/L, *p* = 0.019, respectively), while the concentration of serum λFLC did not differ between MS patients (11.715 mg/L) and controls (13.220 mg/L. *p* = 0.066). Furthermore, the concentrations of κFLC in the CSF differed depending on the types of OCB patterns (ANOVA rang Kruskal–Wallis test: *p* < 0.001, H = 36.472). Post-hoc analysis revealed that the CSF concentrations of κFLC were significantly lower in patients with pattern type 1 (0.300 mg/L) and type 4 (0.936 mg/L) of OCBs in comparison with those with pattern type 2 (2.905 mg/L; *p* < 0.001, *p* = 0.002, respectively) and type 3 (4.400 mg/L; *p* = 0.002, *p* = 0.030, respectively). There were no significant differences in CSF κFLC concentrations between patients with OCB pattern type 2 and type 3 (*p* = 1.000). The concentrations of serum κFLC and λFLC as well as CSF λFLC were similar in all patients irrespective of their OCB pattern type. 

### 3.2. Values of κFLC Index, λFLC Index, κIgG Index, and λIgG Index

**Figure 2:**
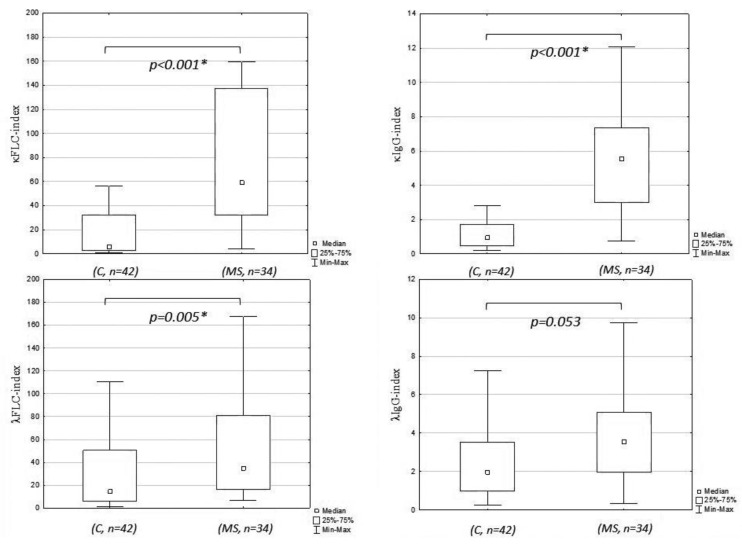
κFLC index, λFLC index, κIgG index, and λIgG index in the study groups. C, control group; MS, multiple sclerosis; *, significant differences in comparison to the controls.

The values of κFLC index, λFLC index, κIgG index, and λIgG index are presented in Table 2. The values of κFLC index, λFLC index, and κIgG index were significantly higher in patients with multiple sclerosis compared to controls, but there were no differences in the λIgG index between the tested groups (Figure 2). The values of κFLC index, λFLC index, and κIgG index differed depending on the OCB pattern type (*p* < 0.001, H = 25.593; *p* = 0.010, H = 11.355; *p* < 0.001, H = 29.608). Post-hoc analysis revealed that the values of the κFLC index and κIgG index were significantly higher in patients with pattern type 2 (median: 58.551, 5.063) in comparison with those with pattern type 1 (5.933, 0.987; p < 0.001 for both) and type 4 (4.166, 0.636; *p* < 0.001 for both). The λFLC index was significantly elevated in patients with pattern type 2 (35.065) in comparison with those with pattern type 4 (7.208, *p* = 0.013). There were also differences in the κFLC index and κIgG index values between patients with pattern type 3 (56.172; 4.503) and those with pattern type 4 (*p* = 0.034; *p* = 0.029, respectively). In addition, the κIgG index was markedly elevated in patients with pattern type 3 compared with those with pattern type 1 (*p* = 0.033). There were no significant differences in the λIgG index between patients with different OCB types (*p* = 0.106, H = 6.123). 

### 3.3. Correlations of CSF κFLC, CSF λFLC, κFLC Index, λFLC Index, κIgG Index, and λIgG Index with Other Parameters Reflecting Pathological Processes in the CNS

The correlations between CSF κFLC, CSF λFLC, κFLC index, λFLC index, κIgG index, and λIgG index with other parameters reflecting pathological processed in the CNS are presented in Table 3. Sprearman’s rank correlation test demonstrated that in the total study group, κFLC, λFLC, κFLC-index, λFLC index, κIgG index, and λIgG index correlated with each other. The CSF concentrations of κFLC and the values of the λIgG index were significantly associated with Q_IgG_. CSF κFLC, CSF λFLC, and κFLC index correlated with Q_IgM_ values, while Q_IgA_ was associated with the values of κIgG index and λIgG index. Furthermore, we observed a negative correlation of Q_Alb_ and patients’ age with κFLC index, λFLC index, κIgG index, and λIgG index. 

### 3.4. Diagnostic Power of κFLC Index, λFLC Index, κIgG Index, and λIgG Index

**Figure 3:**
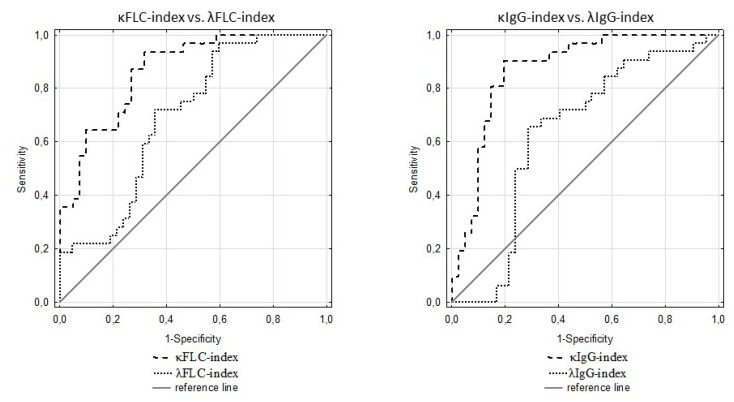
ROC curves for κFLC index, λFLC index, κIgG index, and λIgG index in multiple sclerosis.

The diagnostic usefulness of κFLC index, λFLC index, κIgG index, and λIgG index in multiple sclerosis is presented in Table 2. The κFLC index and κIgG index showed a very high ability to detect MS (sensitivity > 90.00% for both) in comparison to the λFLC index (sensitivity, 71.90%) and the λIgG index (sensitivity, 65.60%). The κIgG index showed the highest ability to exclude multiple sclerosis, with 80.50% specificity and 91.70% negative predictive value. The κIgG index presented the highest diagnostic power (AUC) in the detection of multiple sclerosis in comparison to the λIgG index, κFLC index, and λFLC index (Figure 3).

## 4. Discussion

Multiple sclerosis is an inflammatory neurodegenerative disease characterized by intrathecal IgG synthesis. The detection by isoelectric focusing methods of oligoclonal IgG bands in parallel cerebrospinal fluid and serum samples is actually the gold standard for multiple sclerosis diagnosis [3,12]. However, there are some limitations of OCBs detection, such as still indefinite number of bands in the CSF without corresponding bands in serum defining positive results [13]. OCBs determination is not specific for multiple sclerosis, because the elevated intrathecal synthesis of IgG may occur in other CNS disorders [14]. In addition, OCBs are found in the CSF of about 90% of patients with multiple sclerosis, which means that there is always a group of MS patients without CSF bands [15]. Also, another problem is that isoelectric focusing methods are laborious and often difficult [16]. Taking all this into account, we believe that there is a need to find an additional indicator that can be used to diagnose multiple sclerosis. Therefore, in this study, we tried to define a novel diagnostic model using routinely available laboratory test results to predict multiple sclerosis in patients with symptoms of neurological disorders. 

Firstly, we showed that the mean concentrations of κFLC and λFLC in the CSF and of serum κFLC are markedly elevated in patients with multiple sclerosis. Clearly, these changes in free light chains concentrations may originate from increased synthesis of immunoglobulins, a phenomenon firstly observed in the 1970s–1980s [17,18], or from the fact that light chains are synthesized at a speed more than twice higher compared to fully formed A, M, and G immunoglobulins [19]. Our results are totally consistent with the results obtained by other researchers [20,21,22,23,24,25]. Additionally, our study revealed that the CSF concentrations of κFLC were significantly increased in patients with OCB pattern types 2 and 3, which confirmed intrathecal immunoglobulins synthesis. This may suggest that the concentrations of FLCs in the CSF are highly sensitive and specific for the diagnosis of multiple sclerosis. Our findings of increased free light chains are consistent with those of other studies and support the inclusion of free light chains in our algorithm.

Many studies on the prediction of multiple sclerosis have been published in the past few years. Some studies have proposed a model based on κFLCs and albumin concentrations [16,17,22,23]. Presslauer et al. were the first scientists who developed a formula for the κFLC index and tried to evaluate its diagnostic significance. An index using a cut-off value ≥ 5.9 showed higher sensitivity for the diagnosis of multiple sclerosis than OCBs (96% vs. 80%, respectively) [16]. In our study, using the cut-off proposed by Presslauer et al., the κFLC index showed identical sensitivity with that previously reported, but the specificity for our patients’ group was lower (46.3% vs. 86.0%). Therefore, for further analysis, we decided to use the best cut-off form the ROC. When we used a κFLC index value ≥ 9.4, we achieved a similar sensitivity, but the specificity was still lower than in Presslauer et al. study and equaled 68%. On the other hand, this index value was lower than the cut-off published by Menendez-Valladares et al., which was >10.62 and associated with higher specificity [21]. Despite the differences in the cut-off values and specificity, authors unanimously say that the κFLC index has high sensitivity and probably would avoid OCBs determination in most of patients with suspected multiple sclerosis. 

It is well known that the CSF concentrations of FLCs and IgG are increased in patients with multiple sclerosis. The concentrations of free light chains and IgG have been used for the diagnosis of multiple sclerosis but never combined in a single algorithm. Our study was conducted to develop a new simple model for MS diagnosis using routine laboratory tests to predict this disease in a group of patients with neurological disorders. In our study, these variables were used together for the first time to create the novel κIgG index and λIgG index. We compared the already investigated κFLC index and λFLC index with panels named κIgG-index and λIgG-index combined of FLCs and IgG concentrations. The findings of our study confirmed significant differences in the values of κIgG index and λIgG index between multiple sclerosis patients and individuals with other neurological disorders. We denoted about a 9,5-fold difference of median κIgG index and a 2,4-fold difference of median λIgG index in multiple sclerosis patients in comparison to controls. Moreover, it is important to recognize that our model was developed considering different types of OCBs. Differentiation according to OCBs was chosen because clinically, patients with OCB pattern type 2 are almost always classified as multiple sclerosis patients. We observed that the κIgG index was significantly higher in patients with pattern type 2 in comparison with those with pattern type 1 and type 4. Additionally, only the values of the κIgG index were markedly higher in patients with pattern type 3 than in those with pattern type 4 and type 1, which does not exclude multiple sclerosis. In general, the κIgG index showed higher diagnostic significance compared with the λIgG index. The main factor causing this is probably the dominance of κ free light chains in humans (the normal total κFLC/λFLC ratio is approximately 2:1) [26]. These results indicate that the algorithm combining κFLC with IgG is more valid to evaluate the intrathecal synthesis of immunoglobulins in patients with neurological system disorders than other known algorithms. 

## 5. Conclusions

In conclusion, we showed that a novel, simple κIgG index consisting of four variables combined together (serum κFLC, CSF κFLC, serum IgG, and CSF IgG) can predict the intrathecal synthesis of immunoglobulins and may serve as an additional, potential diagnostic marker for the diagnosis of multiple sclerosis, with a high degree of diagnostic sensitivity and accuracy. The main strength of our study is the use of readily available routine laboratory diagnostics tests. In addition, we examined a group of well-characterized patients including 45% multiple sclerosis patients and 55% controls. The control group in this study was highly heterogeneous; however, the purpose of this study was to determine the value of the κIgG index in the differentiation of multiple sclerosis from other neurological disorders. It is very important to differentiate multiple sclerosis from other neurological diseases, because they often require different treatments. While this study provides novel information about the diagnostic significance of four combined markers in the κIgG-index, in the context of practicality, further studies are required to determine the appropriateness of using the κIgG index as a diagnostic tool for multiple sclerosis in a clinical setting. Studies on larger samples should be performed to validate the quality and precision of the κIgG index. To our knowledge, there are no other studies combining FLCs with IgG concentrations, but we cautiously suggest that, in the future, this parameter could be determined as a complementary diagnostic element to oligoclonal bands determination. 

## Figures and Tables

**Table 1:** Results of laboratory tests for patients with multiple sclerosis and the control group.

	Variable Tested Median (Min–Max Values)											
	Albumin S [g/L]	Albumin CSF [mg/L]	Q_Alb_	IgG S [g/L]	IgG CSF [mg/L]	Q_IgG_	IgM S [g/L]	IgM CSF [mg/L]	Q_IgM_	IgA S [g/L]	IgA CSF [mg/L]	Q_IgA_
**Multiple Sclerosis (*n* = 34)**	43.90 * (33.70–57.40)	187.95 (20.60–487.70)	4.80 (2.77–16.31)	10.72 (6.35–1320.00)	43.19 (3.37–20.47)	5.06 * (2.41–18.60)	1.57 * (0.57–360.00)	1.53 * (0.31–9.43)	1.05 (0.3–4.41)	2.18 (0.81–263.00)	3.47 (0.92–24.20)	1.67 (0.72–15.28)
**Control group (*n* = 42)**	40.00 (17.8–53.90)	196.45 (16.5–815.00)	5.95 (2.15–20.99)	10.00 (4.95–1150.00)	26.65 (2.14–151.72)	3.12 (1.07–19.50)	1.19 (0.35–249.00)	0.58 (0.11–103.00)	0.52 (0.12–8.95)	2.28 (0.02–434.00)	3.84 (0.88–37.20)	1.59 (0.45–15.44)
***p–*value**	0.010 *	0.368	0.123	0.541	0.071	0.002 *	0.047 *	0.004 *	0.002 *	0.965	0.952	0.673

S, serum; CSF, cerebrospinal fluid; *, significant differences in comparison to the controls.

**Table 2:** Values of κFLC index, λFLC index, κIgG index, and λIgG index in multiple sclerosis patients and control group and their diagnostic significance.

	Median	Min	Max	Cut-off from the ROC	Sensitivity [%]	Specificity [%]	PPV [%]	NPV [%]	ACC	AUC
**κFLC-index**										
**MS (*n* = 34)**	59.338 *	4.466	623.565	9.417	93.50	68.30	79.20	69.00	79.20	**0.866**
**C (*n* = 42)**	6.196	0.912	91.081							
**λFLC-index**										
**MS (*n* = 34)**	35.070 *	6.336	792.533	21.446	71.90	64.30	60.50	75.00	67.60	**0.693**
**C (*n* = 42)**	14.450	1.015	157.741							
**κIgG-index**										
**MS (*n* = 34)**	5.660 *	0.751	16.400	1.929	90.30	80.50	77.80	91.70	84.70	**0.871**
**C (*n* = 42)**	0.956	0.216	9.581							
**λIgG-index**										
**MS (*n* = 34)**	3.571	0.330	10.374	3.161	65.6	71.4	63.6	73.2	68.9	**0.632**
**C (*n* = 42)**	1.974	0.241	35.665							

MS, multiple sclerosis; C, control group; FLC, free light chains; ROC, receiver operating characteristic; PPV, positive predictive value; NPV, negative predictive value; ACC, accuracy; AUC, area under the ROC; *, significant differences in comparison to the control group.

**Table 3:** Spearman’s correlations between tested variables in the total study group.

Total Study Group (*n* = 76)	Age	Q_Alb_	Q_IgG_	Q_IgM_	Q_IgA_	CSF κ	CSF λ	κFLC−Index	λFLC−Index	κIgG−Index	λIgG−Index
Q_Alb_											
r	0.403		0.648	0.268	0.778	−0.120	0.049	−0.253	−0.244	−0.472	−0.433
p	<0.005 *		<0.005 *	0.030 *	<0.005 *	0.316	0.678	0.032 *	0.036 *	<0.005 *	<0.005 *
Q_IgG_											
r	0.01	0.648		0.553	0.678	0.405	0.208	−0.197	−0.028	0.034	−0.296
p	0.936	<0.005 *		<0.005 *	<0.005 *	<0.005 *	0.076	0.097	0.81	0.776	0.010 *
Q_IgM_											
r	−0.121	−0.268	0.553		0.547	0.425	0.302	0.333	0.164	0.231	0.015
p	0.331	0.030 *	<0.005 *		<0.005 *	0.005 *	0.013 *	0.007 *	0.185	0.064	0.907
Q_IgA_											
r	0.253	0.778	0.678	0.547		0.01	0.078	−0.034	−0.101	−0.273	−0.335
p	0.031 *	<0.005 *	<0.005 *	<0.005 *		0.936	0.512	0.779	0.397	0.021 *	0.004 *
CSF κ											
r	−0.309	−0.120	0.405	0.424	0.01		0.661	0.802	0.515	0.843	0.372
p	0.007	0.316	<0.005 *	<0.005 *	0.936		<0.005 *	<0.005 *	<0.005 *	<0.005 *	0.001 *
CSF λ											
r	−0.138	0.049	0.208	0.302	0.078	−0.126		0.536	0.72	0.557	0.686
p	0.236	0.678	0.08	0.013	0.512	<0.005 *		<0.005 *	<0.005 *	<0.005 *	<0.005 *
κFLC index											
r	−0.459	−0.253	0.207	0.333	−0.034	0.802	0.536		0.784	0.866	0.495
p	<0.005 *	0.032	0.081	0.007 *	0.779	<0.005 *	<0.005 *		<0.005 *	<0.005 *	<0.005 *
λFLC index											
r	−0.369	−0.244	−0.030	0.164	−0.101	0.371	0.72	0.784		0.659	0.809
p	<0.005 *	0.040 *	0.81	0.185	0.397	<0.005 *	<0.005 *	<0.005 *		<0.005 *	<0.005 *
κIgG index											
r	−0.472	−0.472	0.034	0.231	−0.273	0.843	0.557	0.867	0.659		0.647
p	<0.005 *	<0.005 *	0.776	0.064	0.020 *	<0.005 *	<0.005 *	<0.005 *	<0.005 *		<0.005 *
λIgG index											
r	−0.388	0.432	−0.296	0.015	−0.335	0.372	0.686	0.495	0.809	0.647	
p	<0.005 *	<0.005 *	−0.010 *	0.907	<0.005 *	0.001 *	<0.005 *	<0.005 *	<0.005 *	<0.005 *	

* Statistically significant (*p* < 0.05).
